# Comparison of Collagen Dressing Versus Conventional (Paraffin/Cotton Gauze) for Donor Site Dressing in Split Skin Graft Patients: A Prospective Observational Study

**DOI:** 10.7759/cureus.96890

**Published:** 2025-11-15

**Authors:** Raghavendra Chintha, S. Balakrishnan, Karthikeyan Selvaraj

**Affiliations:** 1 General Surgery, Sree Balaji Medical College and Hospital, Chennai, IND

**Keywords:** collagen dressing, conventional dressing, pain assessment, skin raft donor site, wound healing

## Abstract

Introduction

Effective management of donor site wounds following split-thickness skin grafting (STSG) is crucial for optimal recovery, patient comfort, and prevention of complications. Conventional dressings are often associated with delayed healing, increased pain, and higher infection risk. Collagen dressings possess biological properties that may affect the healing process; however, their clinical advantages over conventional dressings remain to be fully established.

Methods

A prospective observational clinical study was conducted over 18 months (April 2023 - October 2024) at the Department of General Surgery, Sree Balaji Medical College and Hospital. A total of 60 patients undergoing STSG were divided into two groups (n=30 each): one receiving a conventional dressing (paraffin or cotton gauze) and the other a collagen dressing. Wound healing was assessed on postoperative days (PODs) 12, 14, 16, 18, and 22. Pain was evaluated daily for one week using the visual analogue scale (VAS), and other metrics like analgesic usage, infection rates, and patient satisfaction were recorded. Data were analyzed using chi-square and t-tests, with significance set at p<0.01.

Results

The collagen group showed a significantly shorter mean healing time (13.5 ± 1.9 days) compared to the conventional group (16.8 ± 2.1 days, p<0.001). Pain scores were consistently lower in the collagen group (Day 3 VAS: 3.8 vs. 5.9, p<0.001). Infection occurred in one patient (3.3%) in the collagen group versus five patients (16.7%) in the conventional group (p=0.043). Opioid use was notably less in the collagen group (13.3% vs. 33.3%, p=0.048), and patient satisfaction was significantly higher (very satisfied: 46.7% vs. 13.3%, p=0.008).

Conclusion

Collagen-based dressings demonstrated superior performance over conventional dressings (paraffin/cotton gauze) in terms of faster healing, better pain control, reduced infection rates, and higher patient satisfaction. Collagen dressings should be considered a preferred option for donor site management following STSG, particularly where enhanced recovery and patient comfort are priorities.

## Introduction

Skin grafting, particularly split-thickness skin grafting (STSG), is fundamental in treating extensive wounds and burns and for reconstructive procedures. While focus is often on graft uptake, donor site management is an equally critical component of postoperative care. The choice of dressing for these donor sites significantly impacts patient comfort, infection risk, epithelialization rate, and cosmetic outcome [1,2].

Donor site wounds (DSWs) are partial-thickness injuries that heal by re-epithelialization from retained dermal appendages. Conventional dressings such as paraffin gauze remain widely used because of their availability and ease of application; however, they are often associated with discomfort, delayed healing, and suboptimal scarring. These drawbacks have encouraged the exploration of alternative materials designed to enhance the healing environment.

Over the past few decades, several advanced dressings, such as hydrocolloids, alginates, hydrogels, polyurethane films, foams, and biological or biosynthetic materials, have been introduced to promote moist wound healing and improve patient outcomes. Among the biologically active options, collagen-based dressings have attracted attention because of their biomimetic composition and biocompatibility. Collagen, being the predominant structural protein of the extracellular matrix, supports cellular migration, angiogenesis, and granulation tissue formation, all of which are integral to the wound healing process. Previous studies have suggested that moist or bioactive dressings, including those containing collagen, may offer advantages such as faster epithelialization and reduced pain compared with traditional dry gauze [3,4].

Despite evidence favoring advanced options, a lack of standardization and economic constraints often lead to the continued reliance on conventional methods. This prospective observational study was designed to directly compare collagen-based biological dressings against conventional dressings (paraffin or cotton) in a standardized clinical setting to definitively assess their efficacy across critical domains: pain, infection, healing time, and patient satisfaction [5,6].

## Materials and methods

Study design and setting

This was a prospective observational clinical study conducted at the Department of General Surgery, Sree Balaji Medical College and Hospital, Chennai. The study duration was 18 months, from April 1, 2023, to October 1, 2024.

Study population and sampling

The study included 60 patients who underwent split-thickness skin grafting. Patients were randomly allocated into two groups of 30 each: the Conventional Dressing Group and the Collagen Dressing Group.

Inclusion and exclusion criteria

The study included patients presenting with a DSW following STSG harvesting for any clinical indication. Only those who provided informed written consent for participation were enrolled in the study.

Patients were excluded if they were below 18 years or above 65 years of age. Individuals with morbid conditions known to interfere with normal wound healing, such as uncontrolled diabetes mellitus, immunocompromised states, active malignancy, or severe anemia, were also excluded. Additionally, patients with a known hypersensitivity to collagen or its derivatives were not considered eligible for inclusion.

Interventions

Patients were postoperatively managed with either a conventional dressing (paraffin gauze or cotton gauze) or a collagen-based biological dressing (pre-sterilized collagen sheets), applied immediately and maintained until re-epithelialization.

Outcome assessments

Data collection for outcome assessment was carried out using standardized and objective parameters.

Wound Healing

An independent observer, blinded to the treatment groups, evaluated DSWs on postoperative days (PODs) 12, 14, 16, 18, and 22. Healing was graded as follows: Grade 1: Complete epithelialization; Grade 2: Spotty or partial epithelialization; Grade 3: No epithelialization or presence of infection

Pain Assessment

Pain at the donor site was recorded daily during the first postoperative week and weekly thereafter using the visual analogue scale (VAS) ranging from 0 (no pain) to 10 (worst possible pain).

Pruritus Assessment

Patients self-assessed the severity of pruritus weekly from Week 2 to Week 5 using a numeric rating scale ranging from 0 to 10, where 0 indicated no itching and 10 represented the worst imaginable itching intensity.

Analgesic Requirement

The type and duration of analgesic use were documented, with classification into nonsteroidal anti-inflammatory drugs or opioid analgesics as required.

Donor Site Infection

The donor site was examined for signs of infection, and the status was recorded as present or absent based on clinical findings.

Patient Satisfaction

Overall patient satisfaction with the dressing type was evaluated on a five-point Likert scale, ranging from very satisfied to very dissatisfied.

Data analysis

Statistical analysis was performed using manual calculations and statistical software tools. Categorical data were compared using the chi-square test, and continuous variables were compared using an independent t-test. A p-value of <0.01 was considered statistically significant.

Ethical considerations

Ethical approval was obtained before starting the study from the Institutional Human Ethics Committee, Sree Balaji Medical College & Hospital, Chennai, and informed written consent was obtained from all participants. Confidentiality was strictly maintained, with data used solely for research purposes. The study was self-funded by the principal investigator, with no external financial support.

## Results

A total of 60 patients, equally divided into the conventional dressing (n=30) and collagen dressing (n=30) groups, were included in the final analysis. All observed differences between the groups proved to be statistically significant (p<0.05) across the key outcome variables.

Patient demographics and clinical context

The majority of participants were in the 31-45-year age group (40.0%), followed equally by the 18-30-year and 46-60-year age groups (30.0% each). There was a noticeable male predominance (63.3%) in the study population. The most frequent indication for STSG was post-traumatic wounds (36.7%), followed by diabetic foot ulcers (23.3%) and burn wounds (20.0%).

Primary Outcome: Epithelialization and Healing Time

Collagen dressings significantly accelerated the wound healing process. The mean time to complete epithelialization was 3.3 days shorter in the collagen group (13.5 days) compared to the conventional group (16.8 days), which was highly statistically significant (p<0.001). This faster healing was evident on POD 14, where 50.0% of patients in the collagen group achieved complete epithelialization (Grade 1), compared to only 20.0% in the conventional group (Table 1). Conversely, the incidence of incomplete or infected wounds (Grade 3) was higher in the conventional group (26.7% vs. 10.0%, P=0.035) (Table 2).

**Table 1:** Comparison of Primary Healing Outcomes: Conventional vs. Collagen Dressing

Parameter	Conventional Dressing (n=30)	Collagen Dressing (n=30)	p-value
Mean healing time (days)	16.8 ± 2.1	13.5 ± 1.9	< 0.001
Healing time range (days)	14 – 21	12 – 18	
Complete healing (Grade 1) by Day 14	6 (20.0%)	15 (50.0%)	0.029

**Table 2:** Wound Healing Grades on Postoperative Day 14

Healing Grade	Conventional Dressing (n = 30)	Collagen Dressing (n = 30)
Grade 1 – Complete epithelialization	6 (20.0%)	15 (50.0%)
Grade 2 – Partial epithelialization	16 (53.3%)	12 (40.0%)
Grade 3 – No epithelialization/Infected	8 (26.7%)	3 (10.0%)
Total	30	30
p-value	0.035	

Secondary Outcomes: Pain, Analgesic Use, and Complications

Patients in the collagen group reported consistently lower pain scores throughout the first week (e.g., Day 3 VAS: 3.8 vs. 5.9), with this difference being highly statistically significant (p<0.001). This translated to a reduced need for strong medication, as opioid use was significantly lower in the collagen group (13.3% vs. 33.3%, p=0.048). Furthermore, the duration of analgesic use was shorter, with only 10.0% of collagen patients requiring analgesics for 11 days or more, compared to 46.7% in the conventional group. The incidence of donor site infection was markedly reduced in the collagen group (3.3% vs. 16.7%, p=0.043) (Table 3).

**Table 3:** Pain, Analgesic Use, and Complications VAS: Visual analogue scale; NSAIDs: non-steroidal anti-inflammatory drugs

Parameter	Conventional Dressing (n=30)	Collagen Dressing (n=30)	p-value
Mean VAS pain score (Day 3)	5.9 ± 1.0	3.8 ± 0.9	< 0.001
Mean VAS pain score (Day 7)	3.5 ± 1.1	1.4 ± 0.5	< 0.001
Opioid usage (NSAIDs + Opioids)	10 (33.3%)	4 (13.3%)	0.048
Analgesic duration ( ≥ 11 days)	14 (46.7%)	3 (10.0%)	0.0034
Infection rate	5 (16.7%)	1 (3.3%)	0.043

Secondary Outcomes: Patient Experience

A substantially larger proportion of patients in the collagen group reported being "very satisfied" (46.7% vs. 13.3%, p=0.008). Notably, no patients in the collagen group reported dissatisfaction, compared to 30.0% of patients in the conventional group who were neutral or dissatisfied. Patients in the collagen group also experienced less pruritus during late healing, with 56.7% reporting minimal itching (Score 0-2) by postoperative week 5, compared to only 20.0% in the conventional group (Table 4).

**Table 4:** Satisfaction Level

Satisfaction Level	Conventional Dressing (n)	Collagen Dressing (n)	p-value
Very satisfied	4 (13.3%)	14 (46.7%)	0.008
Satisfied	9 (30.0%)	13 (43.3%)	
Dissatisfied/Very dissatisfied	9 (30.0%)	0 (0.0%)	
Minimal pruritus (Score 0–2 at Week 5)	6 (20.0%)	17 (56.7%)	0.012

**Figure 1:**
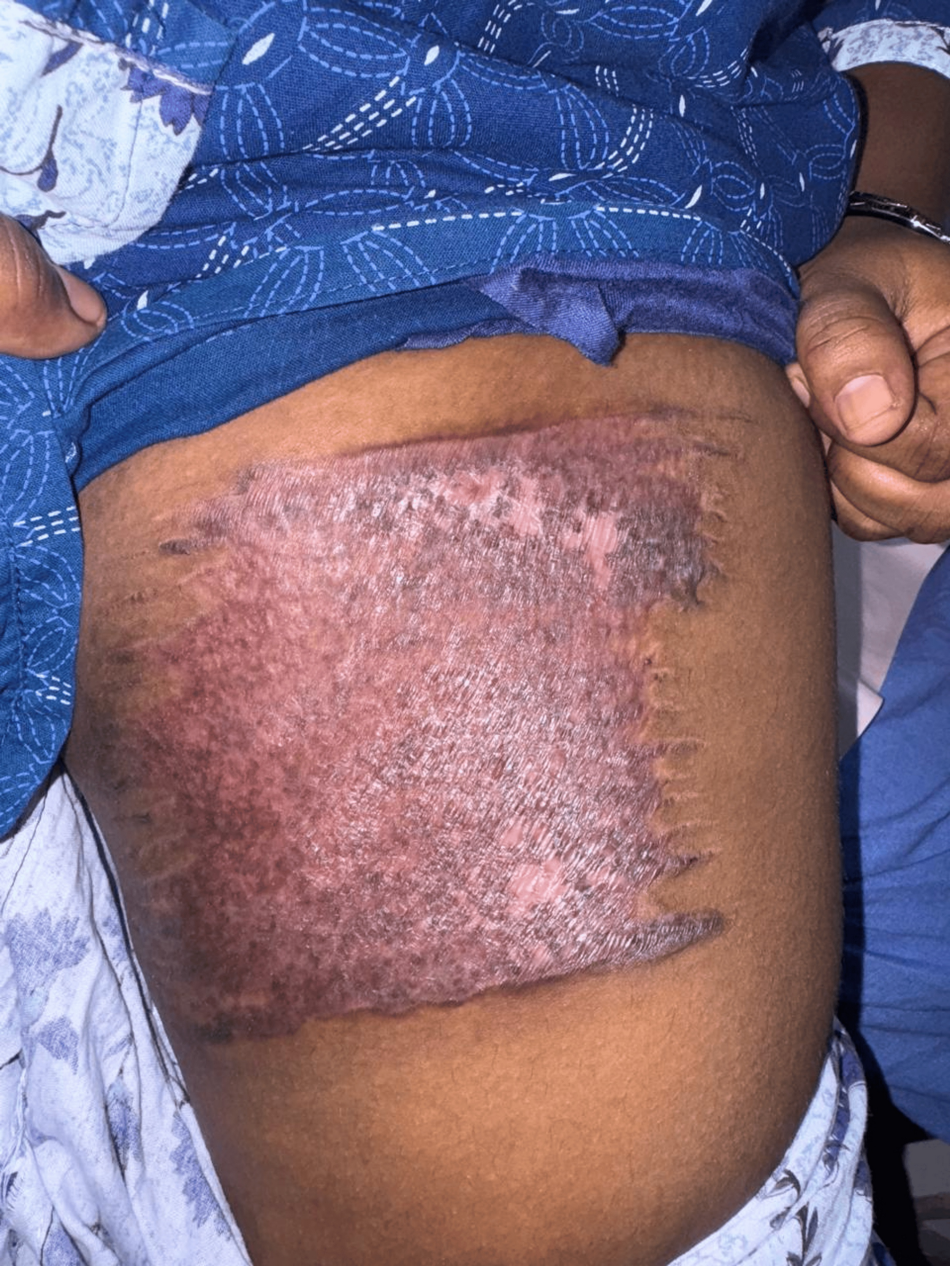
Wound at Day 20 After Surgery Using Collagen Dressing

**Figure 2:**
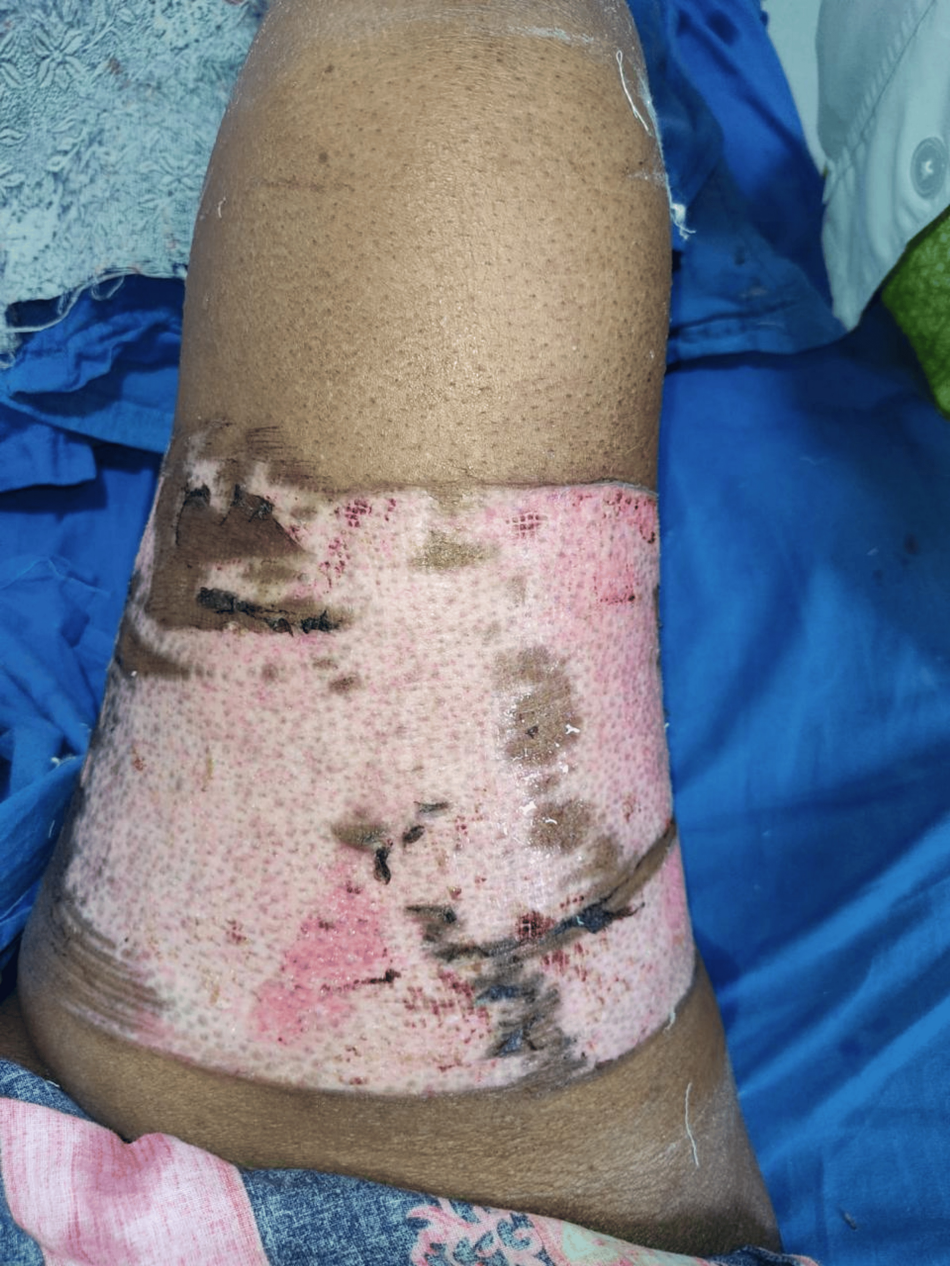
Wound at Day 20 After Surgery Using Conventional Dressing

Better wound condition was seen with collagen dressing (Figure 1) compared to conventional dressing (Figure 2).

## Discussion

The consistency of the observed benefits across all measured clinical parameters presents compelling evidence for the adoption of collagen dressings as a superior alternative to conventional gauze-based methods for the management of STSG donor sites. The findings are particularly significant given the demographic distribution in the study, which reveals a high prevalence of trauma and diabetic ulcer patients-two groups that typically present with impaired healing and increased complication risks. This underscores the clinical relevance of identifying an optimal dressing strategy for such high-risk populations.

Accelerated healing and quality of closure

One of the most significant clinical outcomes observed in this study is the reduction in mean healing time, with the collagen group showing a 3.3-day faster epithelialization compared to the conventional dressing group (p < 0.001). This improvement is not merely a matter of faster closure but reflects a higher quality of healing, as evidenced by the 50.0% rate of complete epithelialization by Day 14 in the collagen group. These results are in line with previous studies, including those by Ramesh et al. [7] (2017) and Sreekumar et al. [8] (2015), which report that bioactive dressings like collagen consistently support faster re-epithelialization, typically within 12 to 14 days, compared to 16 to 18 days for conventional gauze. The underlying mechanism is the collagen matrix’s role as a biological scaffold, promoting cellular migration, capillary ingrowth, and balanced moisture, unlike gauze, which can desiccate or adhere to the wound bed, potentially delaying healing. Teuku et al. [9] (2024) also demonstrated a reduced epithelialization time using biologic agents like honey combined with transparent dressings, reinforcing the principle that bioactive interfaces accelerate re-epithelialization. Thus, the results of the study are consistent with the growing consensus that collagen-based dressings significantly enhance wound closure dynamics.

Enhanced pain control and opioid stewardship

Pain management emerged as another critical area where collagen dressings outperformed traditional methods. A marked reduction in pain was recorded in the collagen group, with Day 3 VAS scores decreasing from 5.9 to 3.8 and sustained lower pain levels noted through Day 7 (3.5 to 1.4). This superior analgesic effect can be attributed to the non-adherent and moisture-retentive nature of the collagen dressing, which minimizes trauma during dressing changes and movement. These findings are consistent with the meta-analysis by Ho et al. [1] (2025), which emphasized the role of moist wound environments in reducing patient discomfort. Similarly, Sinha et al. [10] (2017) in their review of donor site pain interventions emphasized that non-adherent, moist dressings are among the most effective strategies for pain control, rivaling even topical anesthetic interventions in the early postoperative period. The results mirror these conclusions, confirming collagen's superior analgesic profile due to its moist interface, reduced adherence, and bioactive properties. Beyond pain control, the impact on opioid usage is especially noteworthy: the collagen group showed a 60% reduction in opioid consumption (from 33.3% to 13.3%, p = 0.048). This is highly relevant in the current healthcare landscape, where minimizing opioid use is a priority for reducing dependence and enhancing postoperative recovery, aligning with Enhanced Recovery After Surgery (ERAS) protocols. 

Infection prevention and patient satisfaction

Infection rates were significantly lower in the collagen-treated group, with only 3.3% of patients experiencing infections compared to 16.7% in the conventional group (p = 0.043). This difference likely results from the rapid formation of a protective epithelial barrier facilitated by collagen, which helps prevent microbial invasion. Additionally, some evidence suggests that collagen may possess inherent antimicrobial properties, further contributing to infection control, a concept supported by the work of Serebrakian et al. [2] (2018).

The overall clinical benefits also translated into significantly better patient-reported outcomes. A higher proportion of patients in the collagen group reported being “very satisfied” with their treatment (46.7% vs. 13.3%, p = 0.008), and notably, no patients in this group expressed dissatisfaction. In contrast, the conventional dressing group reported more discomfort during the later stages of healing, particularly related to pruritus. By Week 5, only 20.0% of patients in the conventional group reported minimal itching, compared to 56.7% in the collagen group, highlighting a superior healing experience and better patient comfort with collagen-based therapy.

Limitations

This was a prospective observational study, which inherently carries a risk of allocation and performance bias; however, this was minimized through random sampling. While the design helped reduce confounding factors compared to a retrospective approach, it still lacks the rigor of a fully randomized, blinded trial, which would further limit bias. To mitigate observer-related bias, key outcomes such as healing grade assessment were performed by a blinded independent observer. Moreover, as a single-center study, the findings may not be fully generalizable to diverse healthcare settings and patient populations.

## Conclusions

This prospective observational study robustly demonstrated the clinical superiority of collagen-based biological dressings over conventional dressings for STSG donor sites. Collagen dressings yielded statistically significant advantages, including faster wound healing (mean 13.5 vs. 16.8 days, p<0.001), lower pain intensity (p<0.001) with reduced opioid requirements (p=0.048), a lower incidence of infection (3.3% vs. 16.7%, p=0.043), and higher patient satisfaction (p=0.008). These findings suggest that collagen dressings represent an effective and patient-friendly option for STSG donor site management. However, as this was a single-center observational study, further multicentric randomized controlled trials are warranted to validate these results and define collagen dressings’ role in routine clinical practice.
